# Quality of Life in Patients with Benign Non-toxic Goiter after Surgical Intervention: A Systematic Review and Meta-Analysis

**DOI:** 10.1007/s00268-022-06489-x

**Published:** 2022-02-21

**Authors:** Catharina Ihre-Lundgren

**Affiliations:** Karolinska University Hospital, Stockholm, Sweden

It is with great pleasure I have read the systematic review by Chaves et al. with the title “Quality of Life in Patients with Benign Non-Toxic Goiter After Surgical Intervention: A Systematic Review and Meta-Analysis.” [1]

This systematic review studying the health-related quality of life (HRQoL) in non-toxic goiter patients after surgery is comprehensive and well written. The manuscript highlights many of the shortcomings and heterogeneous methodologies that exist in quality of life research in general. The search strategy is adequate and includes the most important databases, and the authors have obviously scrutinized the literature.

Still, it only identified six relevant papers, which could be an effect of the instrument used (ThyPRO); this is rather new and has only recently been translated to non-English languages. Therefore, there may exist a selection, lacking those not published in English journals. Many previous studies have used the well-validated SF-36 instrument for HRQoL, which allows for comparison with the normal population, and which may also adjust for age and gender [2, 3]. SF-36 has been translated into multiple languages. In studies using both SF-36 and ThyPRO, it is obvious that more of the thyroid-specific symptoms affecting HRQoL are better identified with ThyPRO [4].

In this review, the authors have been very attentive in considerations and evaluations regarding bias and have tried to eliminate statistical bias and ensure uniformity by analyzing papers that used the ThyPRO questionnaire only, again the only validated HRQoL questionnaire for benign thyroid disease. They sought to systematically review the differences in HRQoL in patients with benign non-toxic goiter at baseline and 6 months after surgery, using exclusively the ThyPRO questionnaire.

A total of 496 patients were included, and improvement in disease-specific HRQoL was found in all thirteen ThyPRO domains. The results showed that the greatest improvements at 6 months after surgery, compared to baseline, were seen in overall HRQoL, goiter symptoms and tiredness. The complication rate after hemi- or total thyroidectomy is low in patients with a non-toxic goiter, making it even more essential to improve their HRQoL. The forest plot figures are all easy to understand and give the reader a quick overview of the results and the weight of each included studies.

As the authors point out they were not able, due to the small number of included studies, to perform subgroup analyses which could have been interesting. Another interesting question that was not possible to address was voice changes. The ThyPRO questionnaire is not including questions regarding voice changes apart from Recurrent Laryngeal Nerve palsy and its impact on HRQoL.

Although only six studies were included, adding them up still gives us a picture of how patients feel after surgery. The findings of this study can help inform patients, with appropriate indications, who might be hesitant to proceed with thyroid surgery for benign disease. Future studies are still needed, with a high grade of evidence and large population groups, but the groundwork has been established with respect to highlighting the use of disease specific-questionnaire tools and proper statistical analyses to ensure comparability in HRQoL study design.
